# The Association Between JAK2V617F Mutation and Bone Marrow Fibrosis at Diagnosis in Patients with Philadelphia-Negative Chronic Myeloproliferative Neoplasms

**DOI:** 10.5505/tjh.2012.58751

**Published:** 2012-10-05

**Authors:** M. Cem Arı, Deram Büyüktaş, A. Emre Eşkazan, Şeniz Öngören Aydın, Eda Tanrıkulu, Zafer Başlar, A. Nur Buyru, Burhan Ferhanoğlu, Yıldız Aydın, Nükhet Tüzüner, Teoman Soysal

**Affiliations:** 1 İstanbul Training and Research Hospital, Department of Hematology, İstanbul, Turkey; 2 İstanbul University, Cerrahpaşa Medical School, Department of Internal Medicine, İstanbul, Turkey; 3 Diyarbakır Training and Research Hospital, Department of Hematology, Diyarbakır, Turkey; 4 4İstanbul University, Cerrahpaşa Medical School, Department of Internal Medicine, Division of Hematology, İstanbul, Turkey; 5 İstanbul University, Cerrahpaşa Medical School, Department of Medical Biology, İstanbul, Turkey; 6 İstanbul University, Cerrahpaşa Medical School, Department of Pathology, İstanbul, Turkey

**Keywords:** JAK2V617F, Myeloproliferative disease, Bone marrow fibrosis

## Abstract

**Objective:** Bone marrow fibrosis is the second most common complication that causes morbidity and mortality inpatients with Philadelphia-negative myeloproliferative neoplasms (MPNs). The aim of this study was to investigate theassociation between JAK2V617F mutation and bone marrow fibrosis at diagnosis in patients with MPNs.

**Material and Methods:** In total, 149 patients with MPNs were retrospectively evaluated to determine if there was anassociation between the histological grade of bone marrow fibrosis and JAK2V617F mutation.

**Results:** In all, 67.7% of the patients carried the mutated JAK2 gene. The presence of JAK2V617F mutation was notassociated with the occurrence of bone marrow fibrosis (P=0.55) or its grade at diagnosis (P=0.65).

**Conclusion:** Molecular mechanisms or genetic defects other than JAK2V617F may underlie the occurrence of bonemarrow fibrosis in patients with MPNs.

## Abstract (tr)

**Amaç:** Kemik iliği fibrozu, Philadelphia negatif miyeloproliferatif neoplazili (MPN) hastalarda morbidite ve mortaliteye yol açan ikinci en sık komplikasyondur. Bu çalışmanın amacı MPN’li hastalarda tanı anında kemik iliğinde görülen fibroz ile JAK2V617F mutasyonu arasındaki ilişkinin araştırılmasıdır.

**Gereç ve Yöntemler:** Çalışmaya retrospektif olarak dahil edilen 149 MPN tanılı hasta JAK2V617F mutasyonu ile kemik iliğindeki fibroz arasındaki ilişki açısından değerlendirilmiştir.

**Bulgular:** Çalışmaya alınan hastaların %67.7’sinde JAK2V617F gen mutasyonuna rastlandı. Ancak JAK2V617Fmutasyonu ile kemik iliğindeki fibroz varlığı ve derecesi arasında ilişki saptanmadı (sırasıyla, P = 0.55 ve P = 0.65).

**Sonuç:** MPN’de görülen kemik iliği fibrozunun altında JAK2V617F mutasyonu dışında bazı moleküler mekanizma veya genetik bozuklukların yattığı sonucuna varılmıştır.

## INTRODUCTION

Polycythemia vera (PV), primary myelofibrosis (PMF), and essential thrombocythemia (ET) are the 3 classical Philadelphia chromosome-negative chronic myeloproliferative neoplasms (MPNs) that are characterized by clonal proliferation of multipotent hematopoietic progenitor cells. New discoveries concerning the molecular pathogenesis of MPNs have changed the nature of their classification and diagnosis.[1] Janus kinase 2V617F (JAK2V617F) point mutation is a recently identified acquired genetic defect that is present in 95% and 50% of patients with PV and ET/PMF, respectively.[2,3,4] The mutation encodes an inducible tyrosine kinase of the intracellular signaling pathway that promotes myeloid proliferation and differentiation. Bone marrow fibrosis occurs either as a primary disease (PMF) or as a late complication of PV and ET. Fibrosis contributes to morbidity and mortality in patients with MPNs together with additional risk factors.[5,6] Patients usually suffer from fatigue, malaise, weight loss, bone pain, and abdominal distension. The severity of symptoms is associated with the extent of anemia and splenomegaly. Unfortunately, treatment has been palliative and mostly disappointing until recently, consisting mainly of transfusion and other modalities that reduce the size of the spleen. Discovery of JAK2V617F mutation and current progress in the molecule targeted treatment technologies lead to the development of JAK2 inhibitors which seem toyield promising results in selected groups of patients with myelofibrosis and created a certain enthusiasm. But further evidence from randomized studies should be awaited before drawing firm conclusions on the role of JAK2 inhibitors in the management of MPN associated bone marrow fibrosis. It has been reported that there is a possible relationship between the homozygosity of JAK2V617F mutation and the extent of bone marrow fibrosis in patients with PV.[7,8] Tefferi et al. reported that PV patients with homozygous JAK2V617F mutation were more likely to transform into myelofibrosis (23% vs. 2% of patients with homozygous and heterozygous JAK2V617F mutation, respectively). Passamonti et al. also suggested that PV patients homozygous for JAK2V617F are more prone to transformation into myelofibrosis with myeloid metaplasia.[9] The invention of potent JAK2-inhibitor drugs seem to open a new era in the treatment of classic MPNs. The impact of JAK2 inhibitors on bone marrow fibrosis is of clinical importance. As such, the present study aimed to investigate the association between JAK2V617F mutation and bone marrow fibrosis at diagnosis in patients with classic MPNs.

## MATERIALS AND METHODS

**Patients**

We retrospectively reviewed the records of 149 patients with MPNs that were diagnosed and treated between 1996 and 2009 at our institution. Patients were diagnosed according to the international criteria for MPNs that were valid at the time they were diagnosed.[10,11] Bone marrow fibrosis at diagnosis was assessed by an expert hematopathologist and scored according to one of the two grading scales that was in use at the time of pathologic examination as reported in detail elsewhere.[11,12] For practical purposes we merged these two grading scales by rearranging the patients into 4 subgroups with ‘no’, ‘minimal’, ‘moderate’ and ‘marked’ fibrosis. The 2 pathological grading scales and the corresponding 4 subgroups are given in Table 1. JAK2V617F testing has been available at our hospital since 2006; therefore, patients in the study that were diagnosed before 2006 underwent JAK2V617F testing upon entering the study and all the other patients were tested during their initial evaluation. The study protocol was approved by the Ethics Committee of Cerrahpaşa Medical Faculty and was conducted according to the principles of the Declaration of Helsinki. All the participants provided written informed consent to participate in the study. 

**Methods**

JAK2V617F mutation testing was performed via allelespecific polymerase chain reaction (PCR) in all the patients. Into ethylene diamine tetra-acetic acid (EDTA)-containing sample tubes (Greiner Bio-One GmBH, Kremsmünster, Austria), 2 mL of venous blood was collected from each patient via peripheral venipuncture. Using standard techniques, as previously described,[13] DNA was extracted from the blood samples and stored at –70 °C. PCR amplification for the detection of JAK2V617F and visualization of the PCR products were carried out using a commercial assay (Seeplex JAK2 Genotyping Kit). 

**Statistics**

Pearson’s chi-square test and the odds ratios were used to compare and describe the fibrosis occurrence rate between JAK2V617F-positive and negative groups. Statistical analysis was performed using SPSS v.15.0 (SPSS Inc., Chicago, IL, USA). Statistical significance was set at P<0.05.

## RESULTS

Among the 149 patients included, 80 had PV, 37 had ET, and 18 had PMF. Despite exhibiting the classical laboratory and clinical features of MPNs, 14 patients could not be assigned to a specific subgroup and were diagnosed as unclassifiable MPN (uMPN). Patient characteristics are shown in Table 1, 2. In all, 67.7% of the patients carried the mutated JAK2 gene. The frequency of JAK2V617F mutation was highest in the patients with uMPN (85.7%), followed those with PV (80%), PMF (50%), and ET (43.2%). Bone marrow fibrosis (varying in grade) was observed in 107 of the 149 patients at the time of diagnosis (Table 3). 

**Figure 1:**
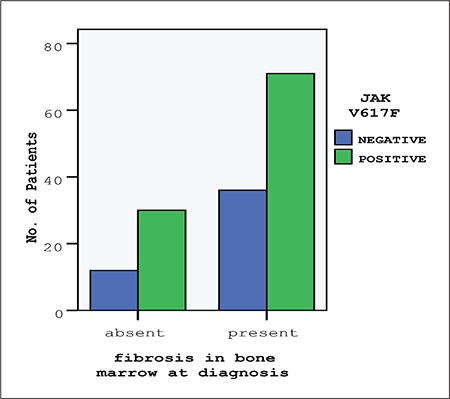
JAK2V617F-positivity and bone marrow fibrosis

**Figure 2:**
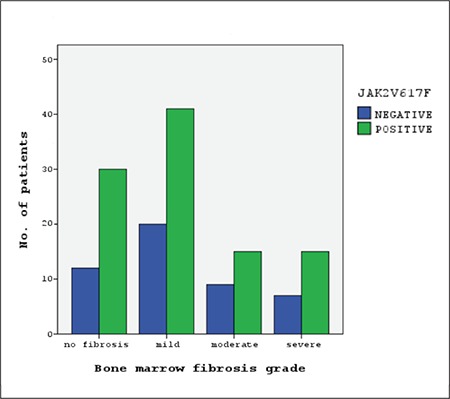
JAK2V617F-positivity according to different grades of bone marrow fibrosis

Bone marrow fibrosis at diagnosis was not associated with the JAK2V617F mutation (P=0.55). The odds ratio for bone marrow fibrosis between the JAK2V617F-positive and JAK2V617F-negative patients was 1.27 (95% CI: 0.58-2.77), i.e. occurrence rate of fibrosis in bone marrow was not increased in JAK2V617F-positive subjects (Figure 1). Analysis of the relation of JAK2V617F to bone marrow fibrosis in PV, ET, PMF, and uMPN subgroups separately did not yield a significant association between fibrosis and the JAK2V617F mutation (Table 4). The grade of bone marrow fibrosis at diagnosis did not differ significantly between patients with and without JAK2V617F mutation (P=0.65) (Figure 2).

## DISCUSSION

The classic MPNs are considered clonal disorders that occur due to some mutations in hematopoietic progenitor cells. Despite new discoveries concerning the molecular mechanism of MPNs, their genetic background is not fully known and requires further investigation. JAK2V617F is an acquired gain-of-function mutation that has been recently described in patients with Philadelphia chromosome-negative MPNs, including primarily PV and ET. The mutated gene encodes the tyrosine kinase called JAK2, which, with other mediators, plays an important role in induction of myeloid cell proliferation and differentiation. JAK2 is an essential component of the intracellular signaling system associated with certain cytokines such as interleukin (IL)- 3, IL-5, and colony stimulating factors and with growth factors like thrombopoietin, and erythropoietin.[14] 

Fibrosis is an essential descriptive component of PMF, and occurs in 5-14% of patients with PV and 15-20% of patients with ET at diagnosis.[11,15,16] In patients with fully developed disease bone marrow is replaced by fibrous connective tissue, causing extramedullary hematopoiesis. Myelofibrosis is the second most common complication in patients with classic MPNs, which leads to cytopenias, splenomegaly, poor quality of life, and reduced survival. [5,17] Intramedullary fibrosis in patients with MPNs occurs through induction of stromal elements in bone marrow via a set of mechanisms. It has been postulated that the mutant JAK2 causes increased proliferation of granulocytes and platelets which in turn, leads to bone marrow fibrosis by producing large quantities of stimulatory cytokines, such as transforming growth factor-beta (TGF-β), platelet-derived growth factor (PDGF), and fibroblast growth factor-beta (FGF-β).[3,7,9] The exact role of JAK2V617F in bone marrow fibrosis remains to be determined. 

JAK2V617F has been reported to occur in 40-91% of patients with MPN-associated myelofibrosis[7]; in the present study 71 of the 107 patients (66%) with bone marrow fibrosis at diagnosis carried the mutation. Considering that the prevalence of JAK2V617F in the MPN patients without bone marrow fibrosis (71%) did not differ significantly from those with fibrosis, we think that there should be other genetic and environmental factors—apart from JAK2V617F that contribute to the deposition of fibrous tissue in the marrow. Passamonti et al. reported that JAK2V617F mutation activates granulocytes and mobilizes CD34 cells, and that the transition of JAK2V617F from heterozygosity to homozygosity could play a role in the progression of PV to post-PV myelofibrosis;[9] however, this has not yet been confirmed. 

The discovery of the association between JAK2V617F and MPNs has facilitated the invention of new targeted treatment strategies and resulted in the development of JAK2 inhibitor drugs for patients with myelofibrosis. A limited number of studies on the use of JAK inhibitors in patients with myelofibrosis reported a dramatic decrease in the size of splenomegaly, but no significant improvement in leukocyte counts, anemia, number of platelets, and bone marrow fibrosis has been observed.[18,19,20] This incomplete resolution of signs and symptoms with JAK2 inhibition supports our view, i.e. JAK2V617F is not the only actor of the scene, there should be other molecular mechanisms and/or mutations which contribute to the development of fibrosis in the bone marrow of patients with MPNs.

Although limited by its retrospective design, the present study clearly shows that the JAK2V617F mutation was not associated with the occurrence of bone marrow fibrosis or its grade at diagnosis. Additional large-scale multi-center studies with MPN patients are required to further delineate the impact of JAK2 and/or other additional factors on the development of bone marrow fibrosis. Studies on newly discovered target-oriented drugs—apart from their contribution to treatment success—may help in uncovering the probable molecular mechanisms of MPNs. 

**Conflict of Interest Statement**

The authors of this paper have no conflicts of interest, including specific financial interests, relationships, and/ or affiliations relevant to the subject matter or materials included.

## Figures and Tables

**Table 1:**
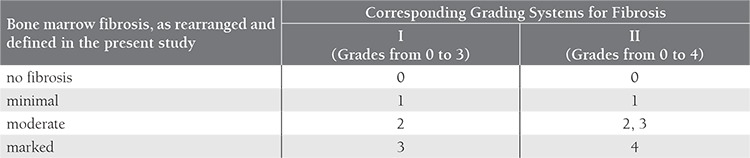
Bone marrow fibrosis

**Table 2:**
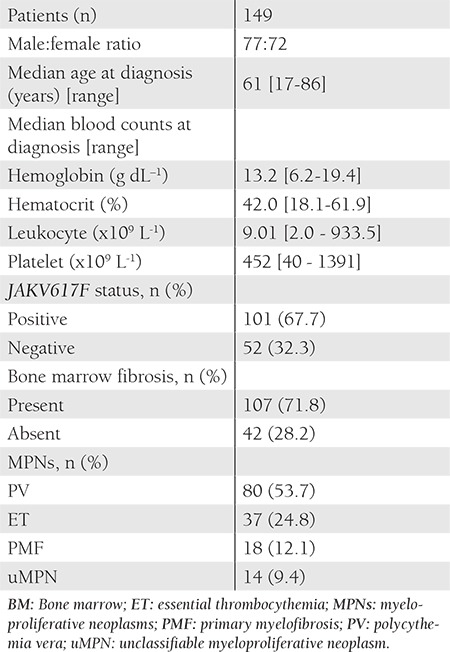
Patient characteristics

**Table 3:**
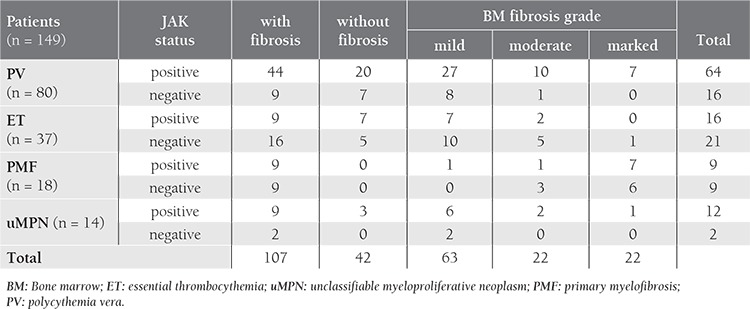
Distribution of MPN patients with and without bone marrow fibrosis, according to JAKV617F status

**Table 4:**
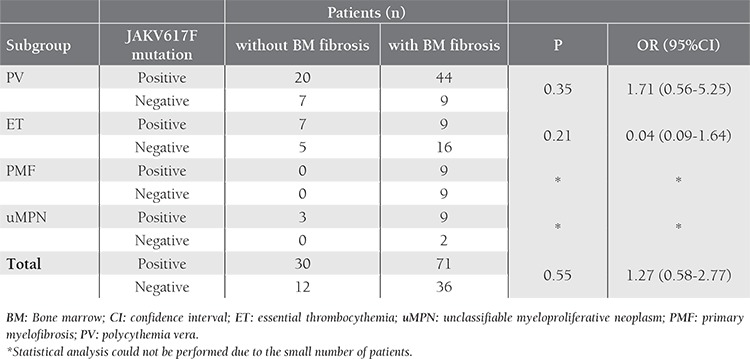
The association between JAK2V617F mutation and bone marrow fibrosis at diagnosis in the MPN subgroups
